# STrack: A Tool to Simply Track Bacterial Cells in Microscopy Time-Lapse Images

**DOI:** 10.1128/msphere.00658-22

**Published:** 2023-03-20

**Authors:** Helena Todorov, Tania Miguel Trabajo, Jan Roelof van der Meer

**Affiliations:** a Department of Fundamental Microbiology, University of Lausanne, Lausanne, Switzerland; University of Wyoming

**Keywords:** algorithm, bioinformatics, cell tracking, image analysis, soil microbiology

## Abstract

Bacterial growth can be studied at the single cell level through time-lapse microscopy imaging. Technical advances in microscopy lead to increasing image quality, which in turn allows to visualize larger areas of growth, containing more and more cells. In this context, the use of automated computational tools becomes essential. In this paper, we present STrack, a tool that allows to track cells in time-lapse images in a fast and efficient way. We compared it to 3 recently published tracking tools on images ranging over 6 different bacterial strains with various morphologies. STrack showed to be the most consistent tracking tool, returning more than 80% of correct cell lineages on average, in comparison to manually annotated ground-truth. The python implementation of STrack, a docker structure, and a tutorial on how to download and use the tool can be found on the following github page: https://github.com/Helena-todd/STrack.

**IMPORTANCE** Automated image analysis of growing prokaryotic cell populations becomes indispensable with larger data sets, such as derived by time-lapse microscopy. The tracking of the same individual cells and their daughter lineages is cumbersome and prone to errors in image alignment or poor resolution. Here, we present a simplified but highly effective tool for non-specialists to engage in cell tracking. The tool can be downloaded and run as a contained script-structure requiring minimal user input. Run times are fast, in comparison to other equivalent tools, and outputs consist of cell tables that can be subsequently used for lineage analysis, for which we offer examples. By providing open code, training data sets, as well as simplified script execution, we aimed to facilitate wide usage and further tool development for image analysis.

## INTRODUCTION

Despite major advances in methodologies describing microbial systems’ states (e.g., the typical ‘o-mics’ tools), there is a fundamental aspect of microbial behavior that is escaping much of our attention. This concerns *in situ* cell division, phenotypic heterogeneity, cell movement, and consequently, local population growth. For most microbiome systems, individual taxa cannot be easily studied in their natural biotic context, nor followed in real-time. Most information, therefore, comes from experiments of reduced complexity, where cell growth can be followed directly by microscopy time-lapse imaging (1). Time-lapse imaging provides direct information on bacterial cell shapes, sizes, and division rates, as well as more complex phenotypes, such as cell movement or stabbing, at the single cell level. It also allows us to visualize the organization of cells into colonies or spatial structures that result from food intake or from interspecific interactions. Relevant information on cell division rates (2), spatial processes (3), or interactions between bacteria (4) can be derived from the images resulting from time-lapse experiments. This requires us to identify single cells in the images, resulting in what is called “segmented masks,” and to track these masks across all time steps correctly.

Several recent methods have been published to facilitate the complex task of tracking cells across time-lapse images (5, 6). Some of these tools integrate both cell segmentation and tracking in a single pipeline (7–9). Since cell segmentation often leads to errors that might have a big impact on the subsequent tracking steps, the tools typically provide a graphical user interface (GUI) that allows users to manually curate results before applying cell tracking. Identified cells are then linked in successive frames based on cell-to-cell distances and similarities. The tracking algorithms are error-prone, and the GUI also offers the possibility to correct tracks manually. Highly interactive methods can, thus, help to perfect tracking results through extensive curation, but they present the drawback of requiring a lot of investment from the users.

Automated tracking tools that can take segmented masks as input have also been recently introduced. For example, Trackmate (10) has been used to track particles (11, 12), and can take object shapes into account when assigning tracks (13). A newer tracking method, Trac^X^ (14), additionally considers the environment of a cell to decide which cells should be linked in successive frames. A third method, DeLTA 2.0 (15), relies on 2 neural networks to identify and track cells in an automated way. These 3 methods are automized, but they require extensive fine-tuning from the user (in the case of Trac^X^), specific Python scripting (e.g., DeLTA), or need to be embedded in commercial software, such as MATLAB (e.g., Trac^X^ and SuperSegger (8)).

The goal of the work presented here was to simplify automated bacterial cell tracking from time-lapse imaging. The tool we developed and tested (called STrack, for Simplified cell Tracking) is implemented in the free Python programming language and runs using one single line of code. We intentionally reduced the parameters that the user needs to define to a minimum of 2 intuitive parameters. The first one defines a restricted search space around a cell, by which the tracking algorithm finds corresponding cells in subsequent images, which drastically reduces the computational running time of STrack. The second parameter defines the division axis, which allows to improve tracking of rod-shaped bacteria specifically, by allowing them to divide only along their cell elongation axis. To facilitate the use of STrack on any operating system, we wrapped it in a docker structure (16). The Docker system prevents conflicts with previously installed packages on one’s computer, and makes STrack’s results more reproducible, as it will return exactly the same results, regardless of any updates of the libraries it relies on.

We first introduce and describe the underlying concept of the STrack algorithm. Then, we present the time-lapse data sets on which we applied STrack, as well as the methods that we used to assess the accuracy of different cell tracking tools. Finally, we compared STrack to 3 of the most recent automated tracking tools: TrackMate, Track^X^, and DeLTA 2.0 (see above), on the same image sets. To compare the tracking performance of these tools, we used expert-generated manual tracking results as ground-truth. We found that STrack on average consistently outperformed the other 3 automated methods on the 22 data sets that we used. Furthermore, we also assessed the tools’ accessibility from a user’s point of view by comparing practical features, such as their running time, free availability, and number of parameters. In the last part, we discuss STrack’s advantages and limitations.

## RESULTS

**FIG 1:**
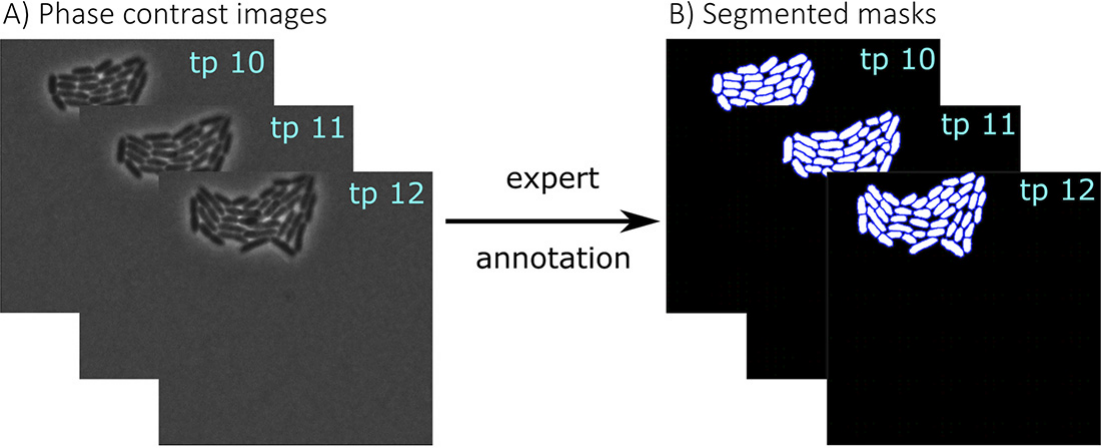
Manual expert segmentation as ground-truth for STrack. (A) Data consisted of phase contrast images capturing growth of bacteria into microcolonies. This figure shows an example of 3 time points (time points 10 to 12, each 10 min apart) of a P. putida microcolony. (B) Identified manually segmented cells (light on dark background).

To compare the performance of STrack to existing tracking tools, we grew 4 bacterial species in pure culture on agarose patches and imaged them using time-lapse microscopy. We limited nutrients in those patches to have a short exponential growth phase, so as to prevent cells from growing in multiple layers. In the resulting phase contrast images, cells were then manually segmented by experts (Fig. 1).

**FIG 2:**
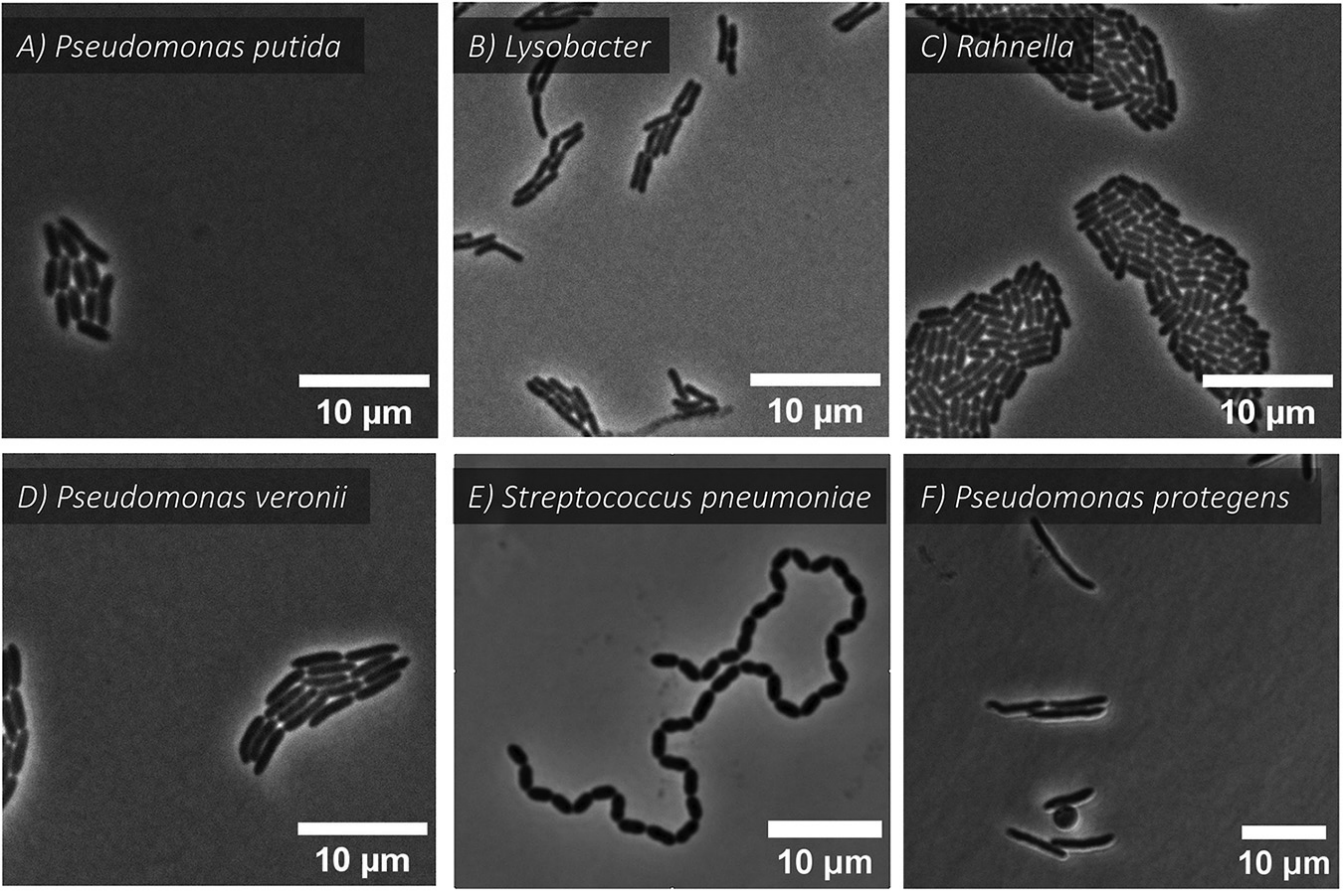
Representative images of 6 bacterial strains included in the tracking analysis and comparison. (A to D) Images of P. putida, *Lysobacter* sp., *Rahnella* sp., and *P. veronii*, respectively. Note the different individual cell morphologies, as well as resulting microcolony shapes. Image in (E) S. pneumoniae (17). (F) P. protegens. The round object in (F) is a spheroblast formed by the activation of tailocins in P. protegens (18).

The different species displayed differences in their growth and cell shapes, with round-shaped densely packed microcolonies (i.e., Pseudomonas putida and *Rahnella*) (Fig. 2A and C), or elongated thinner microcolonies (i.e., *Lysobacter* and *Pseudomonas veronii*) (Fig. 2B and D). Two additional data sets from the literature were included in this study, with further differences in growth patterns that we believed could represent a challenge for cell tracking. The first data set consisted of dividing Streptococcus pneumoniae (17), a coccoid bacterium that grows into long chains (Fig. 2E). The second data set showcased Pseudomonas protegens cell division (18). The induction of R-tailocin formation in Pseudomonas protegens cells results in cell elongation, occasionally followed by the formation of a spheroblast shortly before cell death (Fig. 2F).

**FIG 3:**
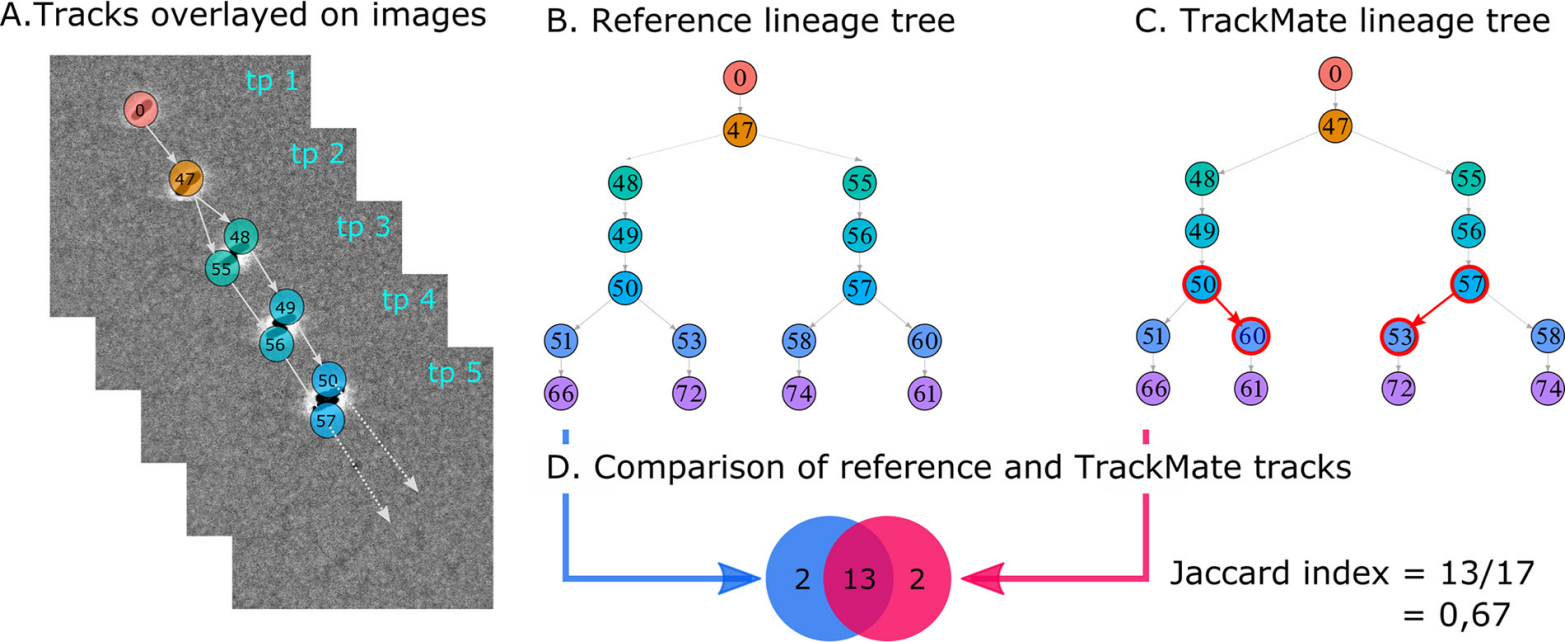
Comparison of tracking results using the Jaccard index. (A) Manually tracked P. putida cells across time points (tp). The tracks are represented as gray arrows and the cells were colored and numbered to be easily compared to (B) the lineage tree corresponding to the tracks from (A). (C) The results of an automated resolved tracking lineage tree over the same images, by TrackMate. Red circles and arrows highlight discordant lineages in comparison to the reference tree. (D) Example of Jaccard index computation. TrackMate returned 13 similar and 2 discordant tracks, resulting in an index equal to the sum of common tracks (=13) divided by the total number of tracks in both methods (=17).

Manually segmented cells were tracked by experts across successive images (Fig. 3A) to build the reference tracks and cell lineage trees for automated tool comparison (Fig. 3B). The automated tracking tools were then compared using the Jaccard index, which represents the proportion of matching and discordant tracks between the results of automated tracking tools and reference tracks (Fig. 3C and D).

**FIG 4:**
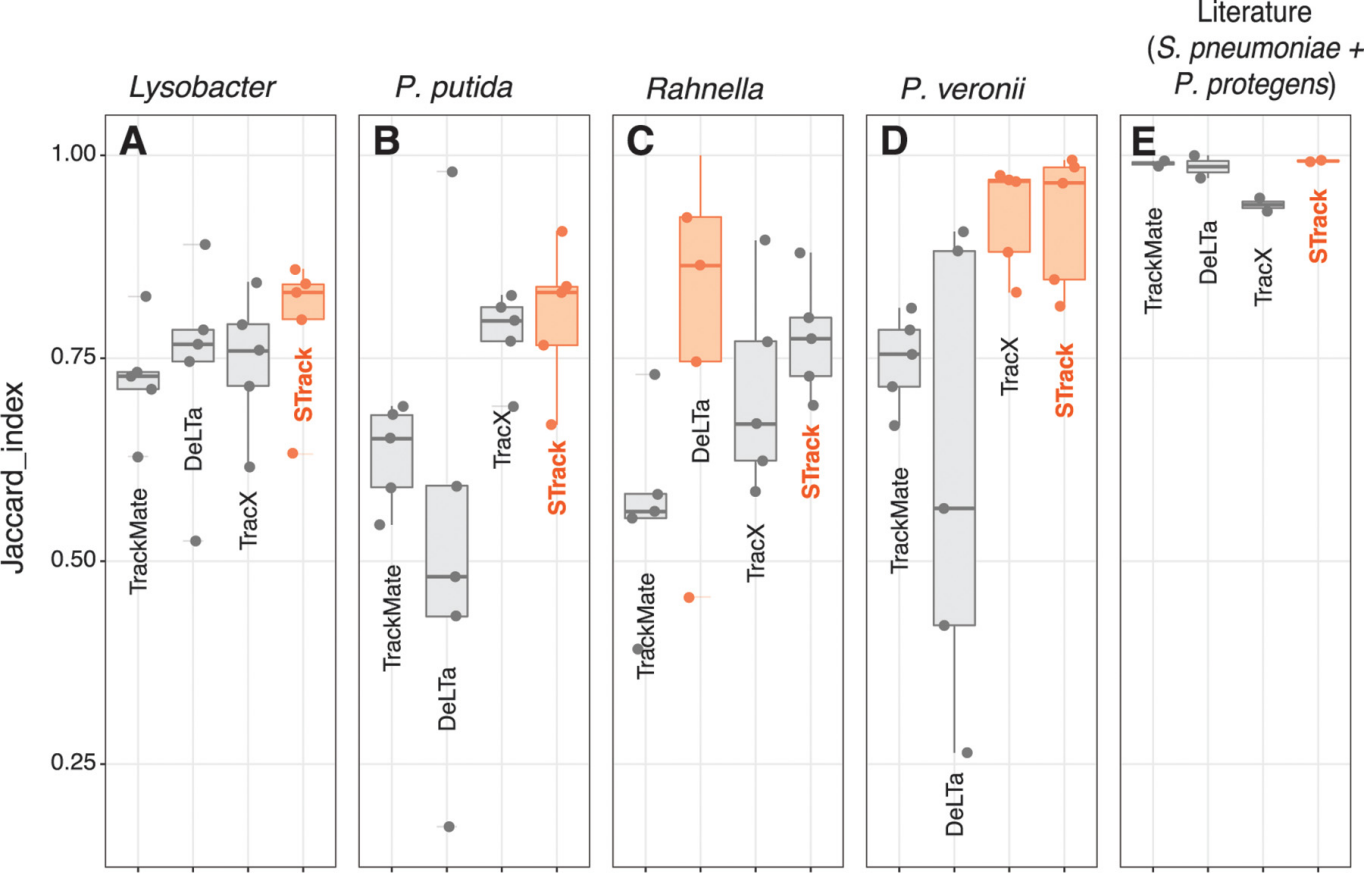
Comparison of 4 cell tracking tools, TrackMate, DeLTA 2.0, Trac^X^, and STrack, on microcolony growth of 6 different bacterial strains. (A to D) Jaccard indices from time-lapse image analysis of *Lysobacter*, P. putida, *Rahnella*, and *P. veronii*, respectively. (E) Tracking results on 2 data sets taken from the literature (corresponding to the images of P. protegens and S. pneumoniae). Boxplots show median (horizontal line) and outlier Jaccard indices (*n* = 4 sets of 22 time-lapse images), overlaid with the individual data points. For each strain, the best performing tools (i.e., with the highest average Jaccard index) are highlighted in orange.

The results of the comparison of STrack and the 3 other tracking tools on the expert-annotated time-lapse series are shown in Fig. 4. STrack and Trac^X^ significantly outperformed TrackMate and DeLTA 2.0 on the P. putida and *P. veronii* series (Fig. 4B and D), but DeLTA 2.0 had a higher average Jaccard index on the *Rahnella* images (Fig. 4C), where STrack was the second-best performing tool. Finally, STrack outperformed the 3 other tools in the images with *Lysobacter* (Fig. 4A).

We then proceeded to a similar comparison of STrack and the 3 other tracking tools on 2 imaging data sets from the literature, consisting of a set with P. protegens images, and another one with S. pneumoniae (Fig. 4E). All tools returned excellent automated tracking results on these 2 data sets, which can be explained by the fact that the P. protegens cells almost did not move or divide, and by the fact that the S. pneumoniae cells were imaged at very short time intervals, which greatly facilitated their tracking. STrack, DeLTA 2.0 and TrackMate had an average Jaccard index between 0.98 and 0.99 on the 2 literature data sets, while the Jaccard indices of Trac^X^ were slightly lower (JI = 0.931 and 0.947).

Across all data sets, the median STrack-JI was higher than that of the 3 others. STrack returned over 83% of correct tracks on average (JI = 0.838), while the second-best tool in this comparative study, Trac^X^, had an average Jaccard index of 0.803, and DeLTA 2.0 and TrackMate, had very similar scores of 0.699 and 0.696, respectively. We also noticed that the variance in individual JI’s of STrack was small, especially compared to TrackMate and DeLTA 2.0 which returned less than 50% of correct tracks on at least 1 data set, while STrack consistently returned over 63% of correct tracks.

**FIG 5:**
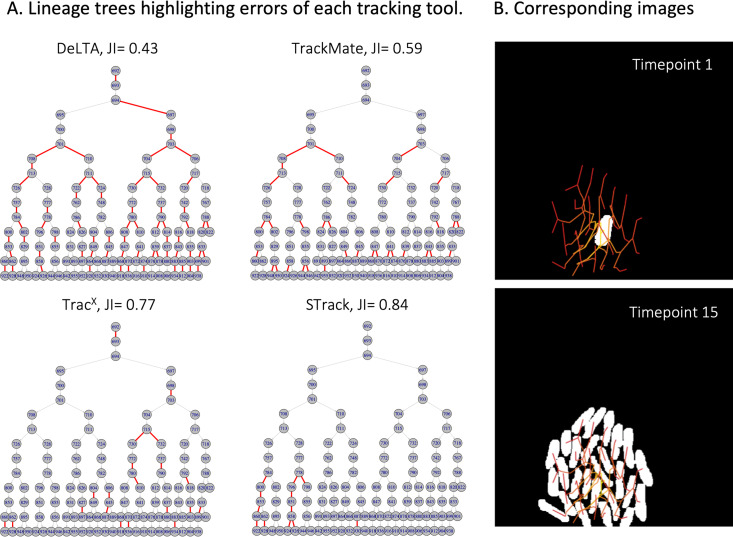
Example of P. putida cell tracking on time-lapse images. (A) Lineage trees obtained with the 4 tracking tools, colored by matches (gray) and mismatches (red) in comparison to manual expert tracking. The calculated Jaccard index (JI) for each comparison is indicated above the corresponding lineage tree. (B) Corresponding cell images of the first and last image of the time-lapse, with manual expert-annotated cell masks represented as white cells. Cell tracks are shown in a red line network overlay on the images.

**FIG 6:**
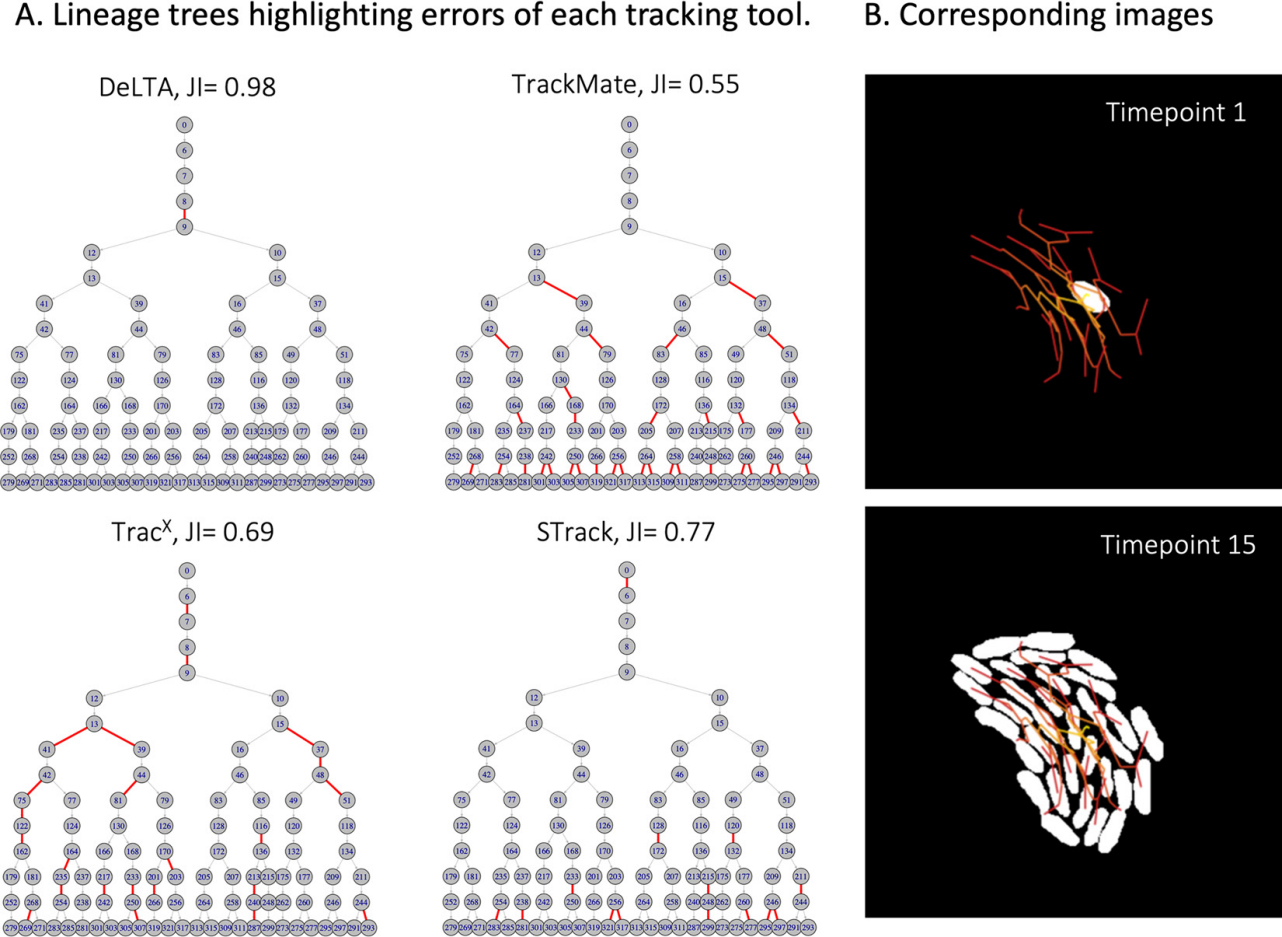
Visual comparison of DeLTA 2.0, TrackMate, Trac^X^, and STrack on a second area of the P. putida time-lapse. (A) Compared to Fig. 5, the global performance of all tools, except DeLTA 2.0, remained relatively similar. (B) Corresponding cell images of the first and last time point of the time-lapse, with manual expert-annotated cell masks represented as white cells. Cell tracks are shown in a red line network overlay on the images.

As an example of STrack’s stability compared to the other tracking tools, we show tracking results on 2 imaged areas of the same time-lapse of P. putida (Fig. 5 and 6). Although these 2 imaged areas come from the same time-lapse experiment, the performance of DeLTA 2.0 drastically changed between the 2 areas. In the first one (Fig. 5A), DeLTA 2.0 returned only 43% of matched tracks, with mismatches spanning all over the lineage tree. However, on the second data set (Fig. 6A), DeLTA 2.0 almost perfectly matched all ground-truth tracks. One obvious difference between the 2 data sets is that one microcolony grew very close to the image border, with some cells exiting the frame (Fig. 5B), while the other microcolony grew at the very center of the images (Fig. 6B). We investigated possible links between the distance of microcolonies to image borders and tracking results, and found a positive correlation for DeLTA 2.0, whose accuracy clearly increased when microcolonies grew further away from borders (Fig. S1). TracX and TrackMate were quite consistent between the 2 imaged areas, returning tracking mismatches that spanned over the whole lineage tree, irrespective of the microcolony position relative to the image borders. STrack also returned relatively consistent results on these 2 data sets, although it performed slightly worse on the data set presented in Fig. 6 (JI = 0.77) compared to the data set in Fig. 5 (JI = 0.84). In both cases, the tracking errors of STrack were mostly localized at later time points down the lineage trees, when cells typically become more difficult to track as the images become crowded. TrackMate systematically performed poorer compared to the other tools, perhaps because it was optimized to track objects with complex shapes (13), whereas the objects here are all very similar in shape and size.

10.1128/msphere.00658-22.1 FIG S1 Analysis of the effect of the microcolonies proximity to image borders on the performance of each tracking tool. The performance is represented by the Jaccard index on the y axis, with a Jaccard index closer to 1 corresponding to a higher performance. The distance of the microcolony to image border is represented on the x axis, in pixels. The association between the Jaccard index and the distance to border was tested for each tracking tool, using the Pearson’s correlation coefficient. The results of these tests are indicated in red boxes. DeLTA 2.0 was the only tool in which we found a significantly positive correlation (correlation > 0 and *P* value < 0.05), indicating that this tool’s performance was significantly lower if the microcolonies grew too close to image borders. Download FIG S1, TIF file, 14.3 MB. Copyright © 2023 Todorov et al. 2023 Todorov et al. https://creativecommons.org/licenses/by/4.0/ This content is distributed under the terms of the Creative Commons Attribution 4.0 International license.

**FIG 7:**
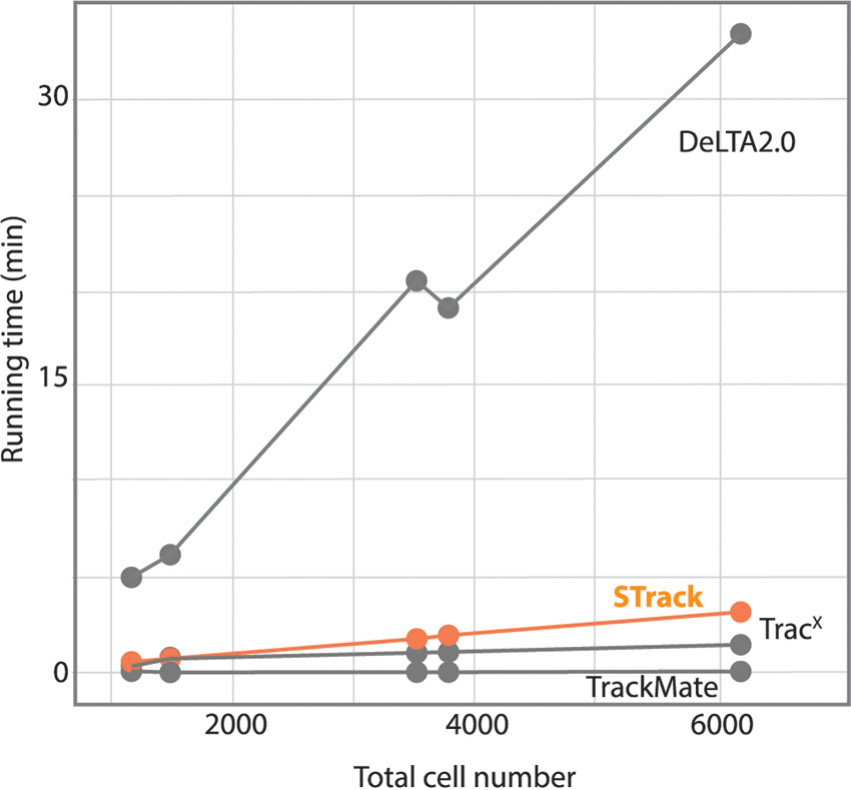
Comparison of the running times of TrackMate, Trac^X^, DeLTA 2.0, and STrack, on 5 time-lapses containing between 1156 and 6172 cells. All tools but one completed cell tracking in less than 1 min on the smallest data sets, and in less than 4 min on the largest data set. DeLTA 2.0, on the other hand, was significantly slower. It took 4 more minutes to run on the smallest data sets, and it needed over 30 min to track cells in the largest data set.

Finally, we assessed the time requirement for tracking as a function of the number of cells per time-lapse. We applied the four tracking tools on data sets containing between 1156 cells and over 6000 cells (Fig. 7 and Table S1). TrackMate was the most efficient tool from a computational point of view, steadily completing the tracking task in a few seconds, regardless of the data size (Fig. 7). The time necessary for Trac^X^ and STrack to complete tracking increased with the number of cells to track, but remained within 4 min for 6000 cells. DeLTA 2.0 was the least efficient of the tools we tested in terms of timing, taking between 5 min in the smallest data set to 33 min in the largest data set.

10.1128/msphere.00658-22.2 TABLE S1 Description of the data sets used to compare tracking running times as displayed in Fig. 7. Download Table S1, TIF file, 4.5 MB. Copyright © 2023 Todorov et al. 2023 Todorov et al. https://creativecommons.org/licenses/by/4.0/ This content is distributed under the terms of the Creative Commons Attribution 4.0 International license.

Different aspects of STrack, TrackMate, Trac^X^, and DeLTA 2.0 that we consider valuable from a user’s point of view are summarized in Table 1. These concern, notably, the tool’s availability in terms of open access, user-friendliness in terms of the number of parameters to be set and necessity to rename files following a specific syntax, computation speed, and accuracy of results. All tools compared in this paper are free, except for Trac^X^ that requires a MATLAB license. Trac^X^ also requires specific file naming (as does DeLTA 2.0), and has 50 parameters that can be tuned, which makes it less user-friendly compared to STrack or DeLTA 2.0. TrackMate was the fastest tool we tested, and the highest overall accuracy in the tracking results was obtained with STrack.

**TABLE 1:** Global comparison of the different cell tracking tools

Tool	Open access	File renaming	No. of set parameters	Computational speed	Accuracy (mean jaccard index)
STrack	Yes	No	2	Moderate	0.838
TrackMate	Yes	No	11	Fast	0.696
TracX	No	Yes	50	Moderate	0.803
DeLTA 2.0	Yes	Yes	0	Slow	0.699

## DISCUSSION

Cell tracking in time-lapse images is important because it allows to gain insight in the real-time behavior of bacteria, on how cells divide, move, or express specific other characteristics (e.g., activation of mobile elements [19] and induction of tailocins [18]). Identifying segmented cells and arranging them into lineages by hand or semi manually quickly becomes untractable. Furthermore, high cell numbers are needed for appropriate statistical comparison of cellular behaviors. There is, thus, a clear need for automated tools that can segment cells correctly, and rapidly group those in relevant tracks with high accuracy. In the tasks we posed ourselves here, namely, deriving cell lineages from dividing cells on surfaces into microcolonies, we found that our tool STrack outperformed 3 other recently published tracking tools. This was tested on time-lapse image series with 6 different bacterial strains, 5 of which had rod-shaped morphologies, and the 6^th^ displaying coccoid cells in elongated chains. STrack consistently returned highly accurate results even in suboptimal images, where microcolonies grew close to image borders and eventually out of frame.

STrack was meant to track segmented objects in time-lapses. The quality of segmentation, thus, plays a crucial role, as mis-segmentations, such as missed cells or merged cells, will inevitably have a deleterious impact on the tool that tries to link these cells between frames. To avoid such segmentation errors, and to generate inputs that would least influence tracking results for optimal comparison of tracking tools, we decided to segment cell contours manually in all the data sets presented in this paper. The tedious task of segmenting cells manually can, however, be replaced by automated segmentation tools (3, 20). As a general rule, we would advise that segmented images are always visually inspected, to make sure that cells are roughly identifiable along a time-lapse series. If many cells remain unassigned, or if they were merged in large aggregates, one should consider improving the segmentation process before proceeding with cell tracking, as tracking poorly segmented cells can only lead to poor tracking results.

The process of cell tracking is rarely isolated. Cell tracking can be performed to gain insight into how cells grow and divide. When coupled with fluorescence cell reporters or stains, cell tracking also allows us to derive information on how gene expression or protein aggregates are transmitted across generations, or appear in sublineages. To derive this type of information, morphological and/or fluorescence information should be computed for every single cell. The morphological/fluorescence profile of single cells can then be merged with the single cell lineage information provided by tools, such as STrack. This type of combined data could then be used to plot morphological or fluorescence information on top of cell lineages, which would allow us to identify biological relevant trends reported by the fluorescence marker.

Many different tools can be used to perform cell tracking, and it can be difficult for a user to decide on tool preference. In this context, STrack can offer a reliable first solution, as it is fast and simple to use, and consistently returns accurate tracking results. For our comparison, we chose not to include interactive tools, such as FAST or Ilastik’s “Tracking with Learning” module (21, 22), which help to achieve excellent tracking results but only after thorough training and curation with high demand of user interference. The main reason for their exclusion was the long, manual handling to achieve tracking. On the other hand, TrackMate, Trac^X^, and DeLTA 2.0 can be applied on segmented masks directly, and only require several parameters to be set (which is not even necessary in the case of DeLTA 2.0). To have a fair comparison, we applied all 4 methods (STrack, TrackMate, Trac^X^, and DeLTA 2.0) on the same data sets, with expert-annotated tracks as reference for the evaluation. Across all data sets, we found that STrack’s results were consistently closest to the manual expert annotation, but we acknowledge that some of the other tracking tools were more precise in certain cases, for example in the case of *Rahnella* microcolony growth. It might, thus, be good practice to test different tracking tools, as their performance might be data set dependent. As an example, we noticed that the effectiveness of DeLTA 2.0 was correlated with the distance of cells to image borders. Making sure to frame cells so that they grow at the center of time-lapse images, or re-training DeLTA 2.0 specifically with microcolonies that grow on image borders, might be solutions to improve its accuracy.

So far, authors of tracking tools have mainly focused on tracking non-motile or low-motile bacteria, assuming that their close cell-neighbor environment is not changing drastically from one frame to the next. This is also the case of STrack, as one of its limitations is that it will link bacteria that are in close vicinity, thus making it inappropriate when bacteria start to move around. In order to tackle the much more complex challenge of tracking motile bacteria in time-lapse images, such as bacteria with a predatory behavior (i.e., Myxococcus xanthus or *Lysobacter* [23]), a new generation of tracking tools will be required. These tools will need to evaluate all possible cell-to-cell combinations to select the most plausible tracking scenarios at the image scale, as opposed to currently looking in the close vicinity of each cell. Alternatively, or additionally, a new generation of tracking tools will also have to consider each bacteria’s movement vector to predict plausible cell movement trajectories. This idea has recently been developed at the image scale (4), but movement vectors will need to be extracted at the single cell level if we want to reach correct tracking, even when bacteria change direction during time-lapse imaging. This will make the task of cell tracking highly computationally challenging.

Analyzing images in an automated way has the advantage to improve reproducibility compared to manual data extraction. Unfortunately, even the same piece of code can still lead to variable results on different computers due to varying package versions and operating systems. STrack overcomes this issue as it is wrapped in a docker structure, in which the versions of all necessary packages are hard-coded. STrack will, thus, more easily operate across different systems, facilitating the generation of consistent results, regardless of the date or the computer on which it is launched. With reliably accurate and stable tools, such as STrack, we aim to contribute to better detection and quantification of interesting biological cell behaviors from imaging, thus facilitating our acquisition of real-time knowledge on bacterial interactions.

## MATERIALS AND METHODS

### Algorithm.

**FIG 8:**
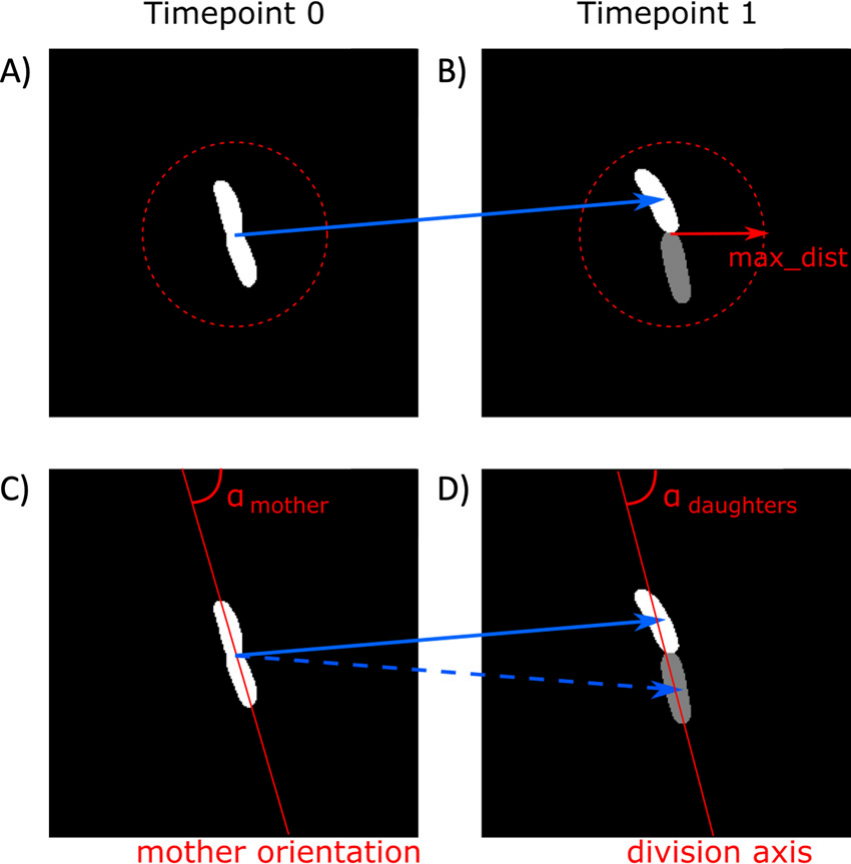
STrack requires 2 user-defined parameters. The first parameter max_dist restricts the search space around a mother cell (A) to look for daughter cells (B). The second parameter max_angle limits the angle between the mother cell’s main orientation axis (C), and the division axis passing through the centers of its 2 putative daughter cells (D). If the angle between these 2 axes is smaller than the user-defined threshold, then STrack will assign tracks (represented as blue arrows) between the mother cell and its 2 daughter cells.

We designed STrack to track segmented objects, that result from manual or automated single cell segmentation, between successive time point images. We adapted the tool to optimize tracking of rod-shaped and coccoid bacterial cells growing in planar conditions with single cell layers, such as produced in microfluidic devices (4) or surface growth chambers (24). Bacterial cells typically divide by elongation, with an elongated mother cell (Time point 0) (Fig. 8A and C) giving birth to 2 smaller daughter cells (Time point 1) (Fig. 8B and D). STrack requires only 2 parameters, which relate directly to the images, to be set by the user: (i) the maximum distance (max_dist) used to look for the progeny of a mother cell (Fig. 8A and B). Setting a maximum distance drastically reduces the time and memory STrack takes to run, rendering it applicable to images containing hundreds of cells. (ii) The maximum angle (max_angle) formed by a mother cell and its 2 daughter cells (Fig. 8C and D). This prevents STrack from allowing a mother cell to divide along an axis that is less plausible from a biological point of view. The max_angle parameter is adjustable to the species under scrutiny, and can be increased to allow the tracking of cells in which division leads to an almost perpendicular angle between the 2 daughter cells.

The algorithm’s pseudocode is provided in Text S1. It iterates over all images of a time-lapse. For every set of 2 successive time points, the algorithm will compute distances and pixel overlap between objects in the first image (we call them mother cells) and objects in the second image (daughter cells) that are in a perimeter delimited by the *max_dist* parameter. Based on the pixel overlap, the algorithm will then assign mother-daughter links (starting with the maximum pixel overlap and then decreasingly looking for cells with less overlap). For a second daughter cell to be assigned to a mother cell, the angle between the mother cell’s main axis and the division axis needs to be smaller than the user-provided *max_angle* parameter. Once all mother-daughter links have been assigned based on pixel overlap, there may remain cells with no mother cells. At this point, the algorithm will switch from pixel overlap to distances between cells to identify any potential missing tracks. It will assign mother-daughter links for increasingly larger mother-daughter cell distances (while still respecting the constraints on the maximum division angle defined above). If the distances become larger than the user-defined *max_dist* parameter, the remaining cells will be assigned to new tracks. This allows to start tracking cells that enter the image in the middle of a time-lapse.

10.1128/msphere.00658-22.3 TEXT S1 Pseudocode of the STrack algorithm. Download Text S1, TIF file, 13.3 MB. Copyright © 2023 Todorov et al. 2023 Todorov et al. https://creativecommons.org/licenses/by/4.0/ This content is distributed under the terms of the Creative Commons Attribution 4.0 International license.

**FIG 9:**
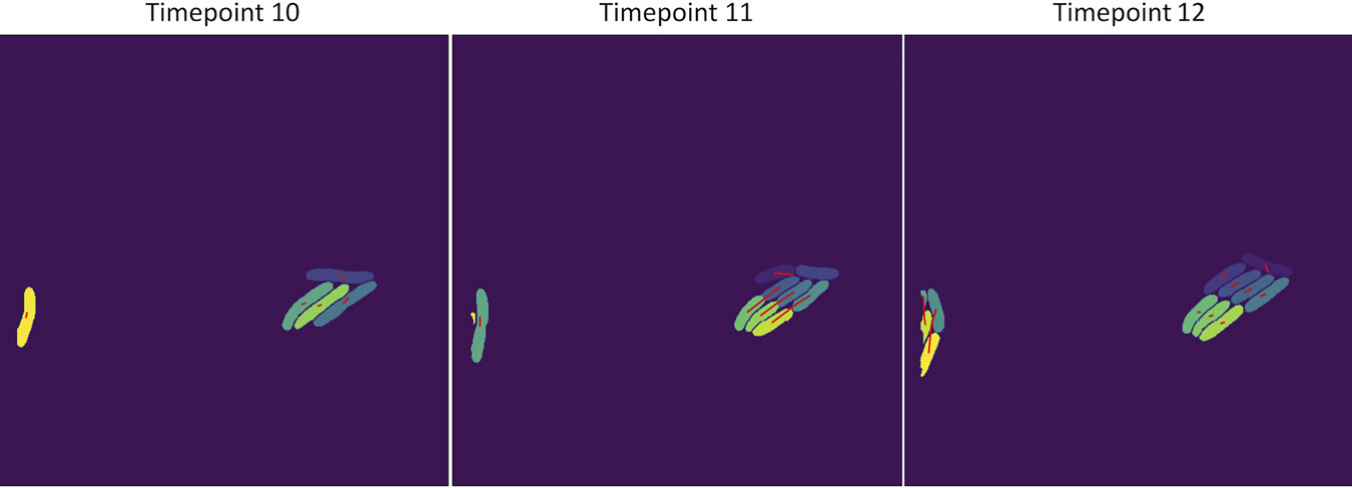
Visual cell tracking validation. For each time point, STrack exports the original cell-mask-image with an overlay of the identified cell tracks. These tracks (represented as red lines) start in the center of mother cells in the image corresponding to the previous time point, and end in the center of their daughter cells in the current image. In this example, STrack identified 5 cells at time point 10, the majority of which divided between time points 10 and 11. These divisions can be seen in the image corresponding to time point 11 as red lines that originate from the centers of their mother cells in time point 10, and lead to their respective daughter cells in time point 11. In this example, STrack even managed to accurately identify the progeny of the 2 cells that were very close to the left image border in time point 11.

The output of STrack is a single CSV-formatted table for every time point. These tables contain tracks from mother cells to daughter cells, including their respective X and Y coordinates, thus allowing to manually verify whether tracks were correctly assigned. Two additional excel-formatted tables are exported, which contain the tracks and the cells from all combined time points, respectively. STrack also returns 1 image per time point, in which the links from the previous to the current image are shown. This facilitates visual checking of the results, as one can directly see whether tracks were correctly assigned between cells or not (Fig. 9). Wrongly assigned tracks can be corrected manually in 2 ways: (i) after visual inspection of the images exported by STrack for every time point, one can manually edit edges in the corresponding csv files. This solution doesn’t require any computational skills, but might be time-consuming. (ii) STrack’s results can be imported into Cytoscape (25), an open-source software for visualizing and editing networks. We provide instructions on how to edit STrack’s results in Cytoscape on the STrack github page: https://github.com/Helena-todd/STrack.

### Time-lapse imaging.

Bacterial cells were grown on miniaturized nutrient agarose surfaces that allow single cells to grow into monolayer microcolonies. Such miniaturized surfaces are enclosed in a black anodized POC (Perfusion Open and Closed) chamber (H. Saur Laborbedarf), which is then mounted on a Nikon ECLIPSE Ti Series inverted microscope coupled with a Hamamatsu C11440 22CU camera and a Nikon CFI Plan Apo Lambda 100X Oil objective, at 22°C. Time-lapse imaging was controlled by a script in MicroManager Studio (v1.4.23). Phase contrast images were taken every 10 to 20 min, depending on the strains, with an exposure time of 30 ms. Cells were imaged on 8 to 10 randomly selected positions per surface, for a duration of 12 to 20 h, resulting in .tif files. We focused on 4 bacterial species isolated from soil: Pseudomonas putida F1 (26), Pseudomonas veronii 1YdBTEX2 (27), *Lysobacter* sp., and *Rahnella* sp. (the 2 latter species coming from Causevic et al. [28]). Cultures were recovered from −80°C stocks, and grown individually on nutrient agar, from which a single colony was transferred and grown in liquid media before being washed, diluted, and inoculated on the microscale-surfaces for imaging. Both pseudomonads were grown with 1 mM succinate as sole carbon substrate in type 21C minimal medium added to the agarose (29). The other 2 strains were cultured with 10-fold diluted PTYG-medium (peptone, tryptone, yeast extract, and glucose), as described by Bakken and Olsen (30).

### Manual image processing.

All time-lapses presented in this paper (including the 2 data sets derived from the literature) were manually analyzed to generate ground-truth segmentation and tracking results. Experts in the field of microbiology manually defined cell masks in these images using the QuPath open-source software for bioimage analysis (31). This resulted in more than a thousand segmented cells over 22 time-lapse data series. The resulting segmented cells were then manually tracked in successive images by the same experts, using the MaMut tracking Fiji plugin (32). This produced the set of manually extracted tracks that we used as ground-truth to compare to the results of automated tracking tools.

### Automated image processing.

In order to assess the computation time necessary for completion of the tracking task by the different tools presented in this paper, we re-used 5 positions of the P. putida and *Rahnella* strains, while taking into account more time points than the ones that had been manually annotated. We purposely selected time-lapses with high cell numbers in crowded images (Table S1). These data sets were used to assess the running times only, not the tracking quality itself. We segmented the cells in these images using the automated cell segmentation tool Omnipose (20). The resulting segmented masks were given as input to STrack, Trax^X^, TrackMate, and DeLTA 2.0, and their computation time was measured on a mac with a i7-9750H processor, 32Go RAM DDR4 running Catalina operating system version 10.15.7.

### Comparison of automated tracking tools.

The cell masks resulting from manual cell segmentation (as described above) were used as input for STrack and the 3 other automated tracking tools that we compared it to. In addition, we included 2 publicly available image data sets from growing Streptococcus pneumoniae (17) and Pseudomonas protegens (18). To track cell objects using TrackMate, we used a procedure from 2021 described by J. W. Pylvanainen on the ImageJ plugins website (33). We did not set any filters on the spots or the tracks, and used the following parameter values: frame-to-frame linking was set to 100 pixels, no gap closing was allowed, and track segment splitting was allowed with a corresponding maximum distance of 50 pixels. In the case of Trac^X^, we contacted the authors who generously shared a script with us (Text S2). For DeLTA 2.0, the authors shared a script on gitlab that we used to inject our manual segmentation results, and only used the tracking feature of DeLTA 2.0 (https://gitlab.com/dunloplab/delta/-/issues/44).

10.1128/msphere.00658-22.4 TEXT S2 MATLAB script to track cells using TracX. Download Text S2, PDF file, 0.04 MB. Copyright © 2023 Todorov et al. 2023 Todorov et al. https://creativecommons.org/licenses/by/4.0/ This content is distributed under the terms of the Creative Commons Attribution 4.0 International license.

The results of STrack, TrackMate, DeLTA 2.0, and Trac^X^ were compared to the manually generated ground-truth cell lineages by quantifying matching and discordant tracks, using the Jaccard index (34). This index reflects the proportion of lineages that were correctly identified by a tool, among the total number of tracks present in the tool’s results and the ground-truth tracks. A Jaccard index equal to 1 would correspond to a perfect match between the tracks returned by a tool and the ground-truth. To visually compare the tracks identified by the 4 tracking tools, they were plotted as a lineage tree or as a red-line network overlaid on cell images using an in-house R script (see Text S3).

10.1128/msphere.00658-22.5 TEXT S3 R script to post-process STrack results. The script allows to plot STrack results as a lineage tree or as a red-line network overlayed on cell images. Download Text S3, PDF file, 0.04 MB. Copyright © 2023 Todorov et al. 2023 Todorov et al. https://creativecommons.org/licenses/by/4.0/ This content is distributed under the terms of the Creative Commons Attribution 4.0 International license.

### Data availability.

The data underlying this article are available on Zenodo: https://doi.org/10.5281/zenodo.7670637.
