# Supplementary figures and images for: STrack: A Tool to Simply Track Bacterial Cells in Microscopy Time-Lapse Images
Source: mSphere. 2023 Mar 20;8(2):e00658-22. doi: 10.1128/msphere.00658-22 (PMC10117057; doi:10.1128/msphere.00658-22)

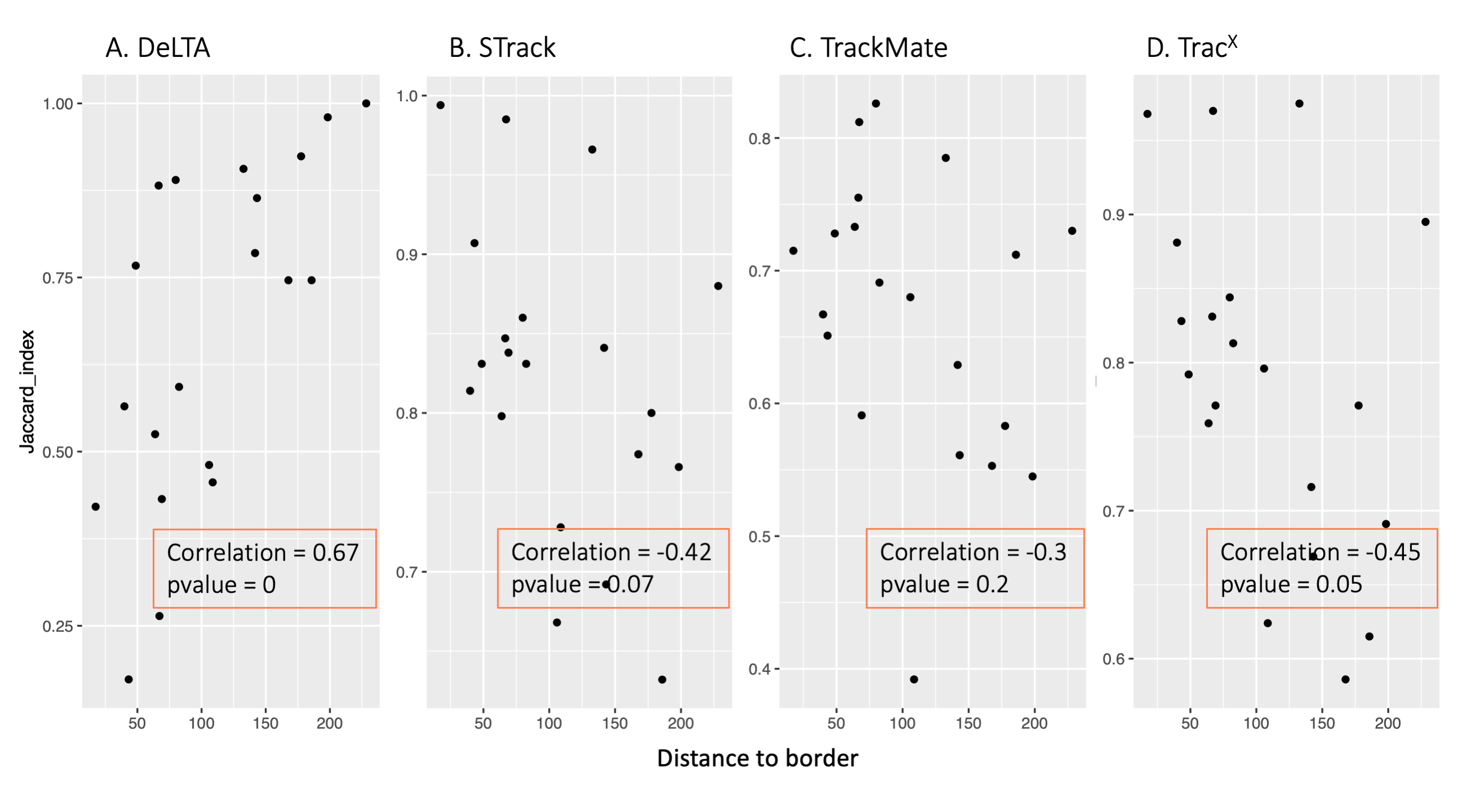

Supplement: FIG S1 [file msphere.00658-22-s0001.tif]

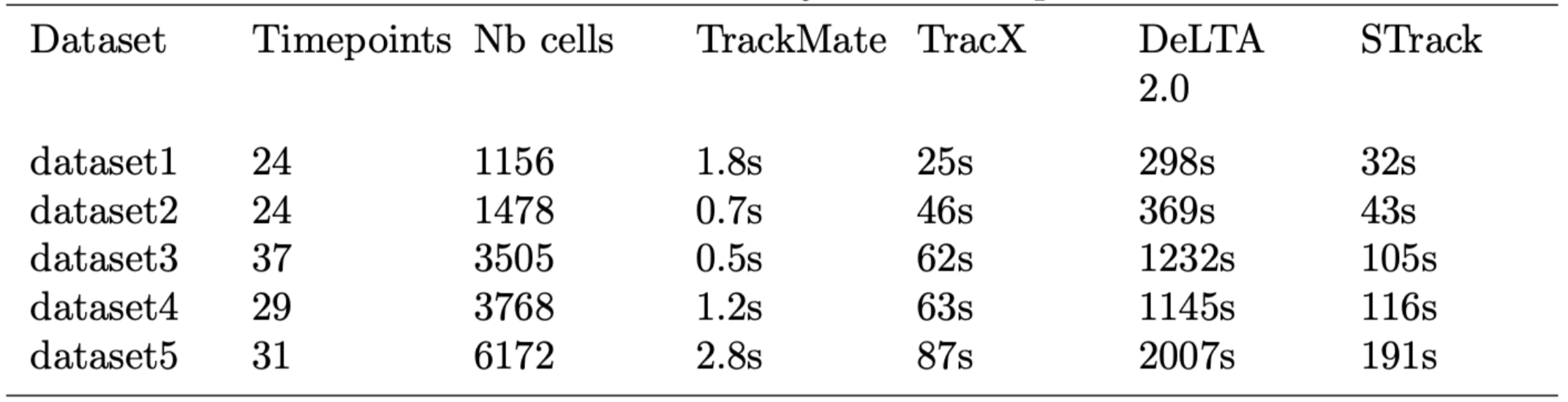

Supplement: TABLE S1 [file msphere.00658-22-s0002.tif]

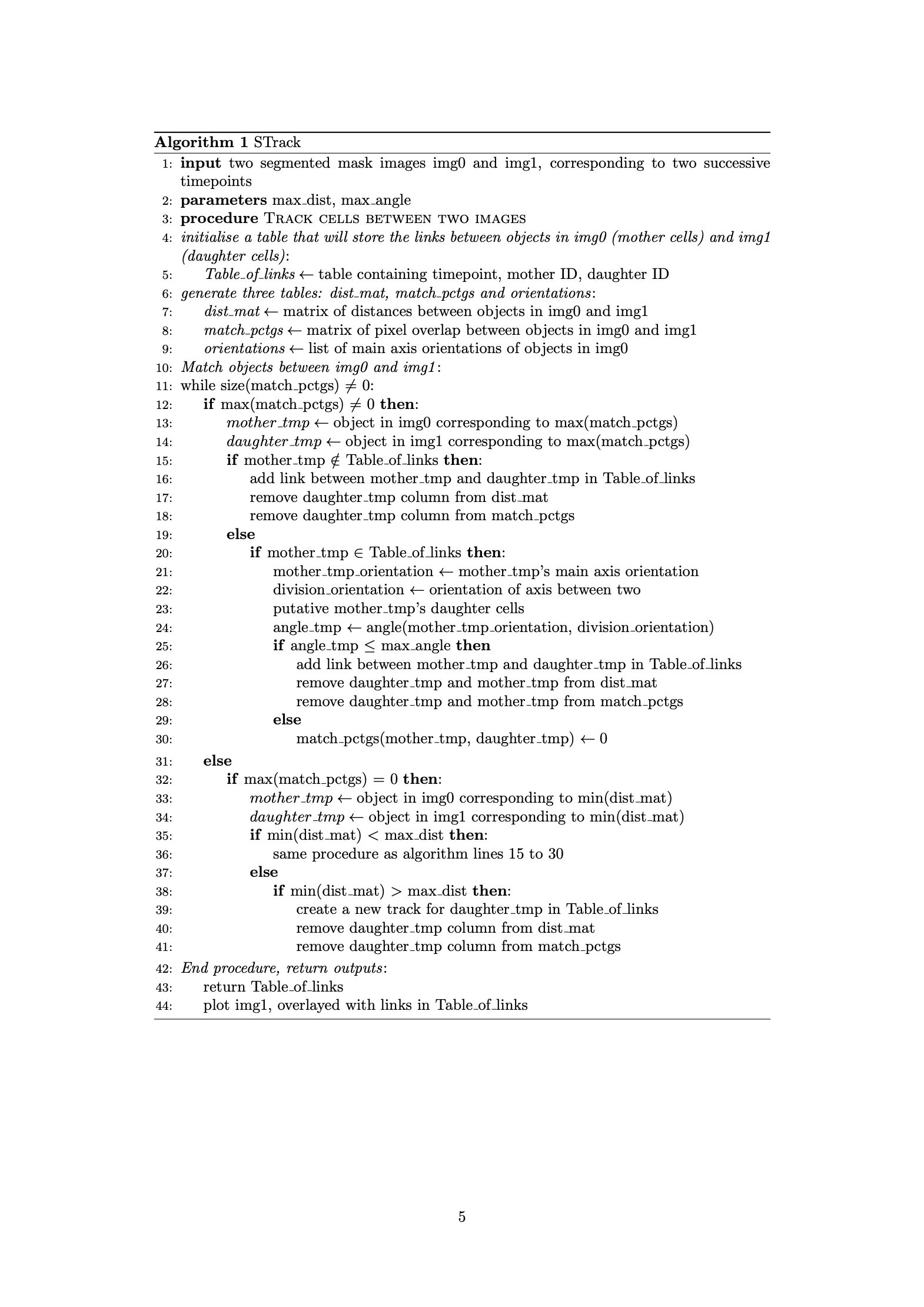

Supplement: TEXT S1 [file msphere.00658-22-s0003.tif]
