# Supplementary material for: STrack: A Tool to Simply Track Bacterial Cells in Microscopy Time-Lapse Images
Source: mSphere. 2023 Mar 20;8(2):e00658-22. doi: 10.1128/msphere.00658-22 (PMC10117057; doi:10.1128/msphere.00658-22)
Supplement: TEXT S2 [file msphere.00658-22-s0004.pdf]

**Supplementary TracX_MATLAB_script:**

```
pathToData = '/Users/helena/Desktop/TracX/'; % change to the directory which contains the 'masks' and 'raw' folder

%% To track a generic segmentation mask (single page tiff file with pixel
% belonging to a cell labeled with a increasing number or binary mask) we
% first have to convert the data and quantify potential signals.
% Note: this step will require the parallel computing toolbox and take some
% time to quantify all images.
Tracker = TracX.Tracker();
imagePath = fullfile(pathToData, 'raw'); % Path to raw images.
segmentationPath = fullfile(pathToData,  'masks'); % Path to segmentation masks.
segmentationFileNameRegex = 'mask_position3_*'; % File identifier for the segmentation masks
imageFileNameRegex = 'BF_*'; % The Brightfield or phase contrast image.
fluoTags = {}; % The channels for signal quantification
Tracker.prepareDataFromSegmentationMask(imagePath, ...
    segmentationPath, segmentationFileNameRegex, ...
    imageFileNameRegex, 'fluoTags', fluoTags)
clear Tracker

%%
% Configure a new tracking project
projectName = 'TracX_formated'; % Project name
fileIdentifierFingerprintImages = 'BF'; % Image identifier for Brigthfield images;
fileIdentifierWellPositionFingerprint = []; % Well position identifier if multiwell experiment.
fileIdentifierCellLineage = ''; % Image identifier for the Cell Lineage reconstruction (i.e bud neck marker).
imageCropCoordinateArray = []; % Empty if no crop has been applied in CellX, add CellX cropRegionBoundaries
coordinates otherwise (from CellX_SCerevisiae_Parameter.xml).
movieLength = 58; % Number of timepoints to track
cellsFilePath = fullfile(pathToData, 'masks'); % Path to segmentation results (CellX Style).
imagesFilePath = fullfile(pathToData, 'raw'); % Path to raw images.
cellDivisionType = 'sym'; % Cell division type.

% Create a tracker instance and a new project
Tracker = TracX.Tracker();
Tracker.createNewTrackingProject(projectName, imagesFilePath, ...
        cellsFilePath, fileIdentifierFingerprintImages, ...
        fileIdentifierWellPositionFingerprint, fileIdentifierCellLineage, ...
        imageCropCoordinateArray, movieLength, cellDivisionType);
Tracker.revertSegmentationImageCrop() % Reverts the potential image crop applied by CellX to map the cell
coordinates to the full image.

%% Test default tracking parameters
% Optionally tune tracking parameters for better results
Tracker.configuration.ParameterConfiguration.setNeighbourhoodSearchRadius(60); % Increase the neighbourhood to
include enough cells
Tracker.configuration.ParameterConfiguration.setMaxCellSizeDecrease(0.9); % Penalize cell shrinkage to initiate new
tracks after division
Tracker.configuration.ParameterConfiguration.setMaxMajorAxisRotation(30); % Pinalize rotations over 30 degres of
the cells major axis
Tracker.configuration.ParameterConfiguration.setMaxTrackFrameSkipping(1);
Tracker.configuration.ParameterConfiguration.setUsedFunctionsForCostMatrix([1,1,1,1]); % Include the cost for the
orientation / rotation of the major axis and frame skipping
% Dry run to test the tracking parameters
Tracker.testTrackingParameters([1,58]) % Track from frame 1 to frame 15 for testing.

%% Save the tracking results
Tracker.saveCurrentTrackerState() % Saves the tracker state as mat file (to continue work anytime later)
Tracker.saveTrackingProject() % Saves the tracking project.
Tracker.saveTrackerResultsAsTable() % Saves the tracking results as one column seperated table for further
analysis.
Tracker.saveTrackerProjectControlImages('isParallel', false, 'maxWorkers', 6) % Save additional control images to
inspect the sucess of the tracking.

%% Run lineage reconstruction
Tracker.runLineageReconstruction('symmetricalDivision', true, 'writeControlImages', false);

%% Save cell cycle data
Tracker.saveTrackerCellCycleResultsAsTable(pathToData, pathToData, 'resultFileName', 'CellCycleSummary')

%% Print the cell cycle phase information as table and save it
Tracker.lineage.cellCyclePhaseTable
Tracker.saveTrackerCellCycleResultsAsTable()
```
