# Supplementary material for: STrack: A Tool to Simply Track Bacterial Cells in Microscopy Time-Lapse Images
Source: mSphere. 2023 Mar 20;8(2):e00658-22. doi: 10.1128/msphere.00658-22 (PMC10117057; doi:10.1128/msphere.00658-22)
Supplement: TEXT S3 [file msphere.00658-22-s0005.pdf]

Supplementary STrack postprocess R_script:

```
## This script allows to read STrack results and generate two PDF plots:
## 1) a lineage tree
## 2) a cell tracks plot displaying the tracks using the x-y coordinates of cells

## the two PDF plots will be outputed in the STrack results folder,
## that should be provided by the user in line 13 of this script.

library(openxlsx)
library(tidyverse)
library(igraph)
library(cowplot)

# provide path to STrack results
data_path <- "/Users/helena/Desktop/STrack/"

#################################################
######    Plot and save the lineage tree   ######
#################################################

# read the csv file containing STrack's results for all timepoints
# and use it to generate a graph
edges_df <- read.xlsx(paste0(data_path, "complete_tracking_table.xlsx"),
                     rowNames = FALSE) %>%
  mutate("from" = Mother_mask, "to" = Mask_nb) %>%
  select(from, to, colnames(.)[-which(colnames(.) %in% c("from", "to", "Unnamed:.0"))]) %>%
  filter(from != to) # remove loops on single nodes

gr <- igraph::graph_from_data_frame(as.matrix(edges_df), directed = TRUE)

# Generate a pdf file containing the lineage tree
pdf(paste0(data_path, "lineage_tree.pdf"), height = 10, width = 10)
plot(gr, layout=layout_as_tree, vertex.size = 5, edge.arrow.size=0.5)
dev.off()

#####################################################
######    Plot and save the cell tracks plot   ######
#####################################################

# note: this piece of script was adapted for images of 512*512 pixels
# please change the hardcoded values to adapt them to larger/ smaller images
my_plot <- ggplot(edges_df, aes(x=Centroid_x_mother, y=-Centroid_y_mother,
                                xend=Centroid_x, yend=-Centroid_y)) +
    geom_segment(color = "red") +
    xlim(0,512) + ylim(-512, 0) +
    theme_void() +
    theme(legend.position="none")

pdf(paste0(data_path, "cell_tracks_plot.pdf"), height = 6, width = 6)
# the user can provide a png image to plot in the background
p1 <- ggdraw() +
  draw_image(paste0(data_path,"tracking_figure_time1.png")) +
  draw_plot(my_plot, scale = 0.8, width = 1.05, hjust = 0.013,
            height = 1.05, vjust = 0.03)
plot(p1)
dev.off()
```
